# P-1479. Differential Uptake of Zoster and COVID-19 Vaccines in a System without Financial Barriers: A Multivariable Analysis of Race, Demographics, and Provider Factors

**DOI:** 10.1093/ofid/ofaf695.1665

**Published:** 2026-01-11

**Authors:** Nicholas Mielke, Raul Isern, Nirpeksh Jain, Nansea Ji, Kelsey Witherspoon, A Corey Yeates, Jennifer Zimmerman, Marvin J Bittner

**Affiliations:** Creighton University School of Medicine, Omaha, NE; Creighton University, Omaha, Nebraska; Creighton University School Of Medicine, Elkhorn, NE; Creighton University School of Medicine, Omaha, NE; Creighton University School of Medicine, Omaha, NE; Creighton University, Omaha, Nebraska; Creighton University, Omaha, Nebraska; Creighton University School of Medicine, Omaha, NE

## Abstract

**Background:**

A quality improvement project examining vaccine uptake in a primary care clinic had found a racial disparity in zoster vaccination but not in initial COVID-19 vaccination. This finding was notable since patients in this setting had access to vaccines at no cost, mitigating a barrier to uptake. We sought to identify factors contributing to this finding by analyzing a sample of this population.

**Figure:**
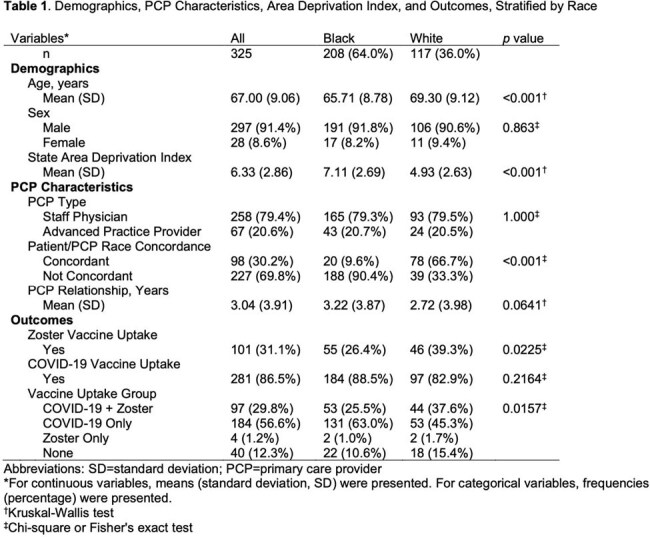

**Figure:**
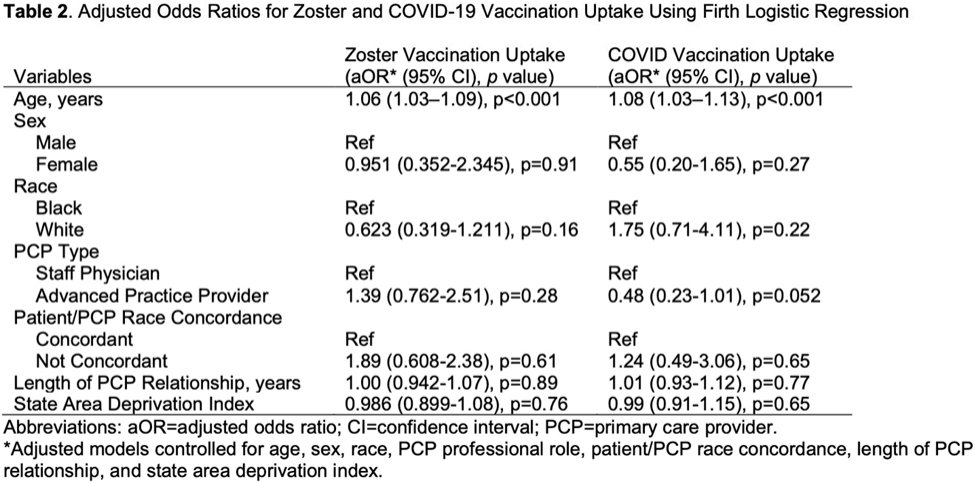

**Methods:**

This retrospective cohort study randomly selected 400 White and 400 Black adults who visited a primary care clinic at an academic medical center, where there is no charge for vaccines, between October 1, 2020, and July 5, 2021. The analysis was limited to patients residing in the hospital’s county and seen by staff physicians or advanced practice providers. Demographic data, primary care provider (PCP) characteristics, and vaccination records were studied.

**Results:**

325 patients met the inclusion criteria: 208 (64.0%) identified as Black and 117 (36.0%) as White. The average age was 67 years and 91.4% were male. Black patients had higher area deprivation index (ADI) scores (p< 0.001). Zoster vaccine uptake was lower among Black patients compared to White patients (26.4% vs. 39.3%; p=0.0225), but COVID-19 vaccine uptake was similar (88.5% vs. 82.9%; p=0.2164). In Firth logistic regression models adjusted for age, sex, race, PCP type, PCP/patient race concordance, length of PCP relationship, and ADI, older age was associated with higher adjusted odds of both zoster and COVID-19 vaccine uptake (both p< 0.001). Race was not significantly associated with uptake of either vaccine after adjustment (both p >0.05).

**Conclusion:**

In a patient population with access to primary care in a clinic without charges for vaccines, Black patients had lower zoster vaccine uptake. However, this difference was not statistically significant after adjusting for demographic and provider-related factors as well as ADI.

**Disclosures:**

Marvin J. Bittner, MD MSc, Bavarian Nordic: Honoraria|GSK: Advisor/Consultant|Sanofi: Honoraria|Valneva: Advisor/Consultant

